# Efficacy of silicone soft reliner on the obturator prosthesis after maxillectomy for oral malignant tumors: A single‐arm prospective interventional study

**DOI:** 10.1002/cre2.326

**Published:** 2020-09-08

**Authors:** Souichi Yanamoto, Sakiko Soutome, Maho Murata, Akiko Kawakita, Erika Yamaguchi, Kazuhiro Yoshida, Tadafumi Kurogi, Shinichiro Kuroshima, Hiroshi Murata, Takashi Sawase, Masahiro Umeda

**Affiliations:** ^1^ Department of Clinical Oral Oncology Nagasaki University Graduate School of Biomedical Sciences Nagasaki Japan; ^2^ Department of Oral Health Nagasaki University Graduate School of Biomedical Sciences Nagasaki Japan; ^3^ Department of Prosthetic Dentistry Nagasaki University Graduate School of Biomedical Sciences Nagasaki Japan; ^4^ Department of Applied Prosthodontics Nagasaki University Graduate School of Biomedical Sciences Nagasaki Japan

**Keywords:** masticatory performance, maxillectomy, obturator prosthesis, oral health‐related quality of life, prospective interventional study, silicone soft reliner

## Abstract

**Background:**

There is insufficient evidence for the efficacy of silicone soft reliner on the obturator prosthesis after maxillectomy for oral malignant tumors.

**Objective:**

To verify the efficacy of silicone soft reliner on the obturator prosthesis after maxillectomy, by evaluating masticatory performance and quality of life (QoL).

**Methods:**

This was a single‐arm prospective interventional study, verifying the efficacy of silicone soft reliner (GC RELINE II®) on the maxillary obturator prosthesis. Data were obtained from a comparison of the endpoints after 14 days of continuous use of acrylic and silicone soft‐lined prostheses. The primary endpoint was masticatory performance. The secondary endpoints were occlusal performance and oral health‐related QoL (OHRQoL). The masticatory performance, occlusal performance, and OHRQoL were assessed by glucose concentration, maximum bite force, and the Japanese version of Oral Health Impact Profile (OHIP‐J49), respectively.

**Results:**

This study included five patients (two males, three females), aged between 71 and 88 years, with a median of 74 years. The median of glucose concentration indicated a statistically significant improvement between the acrylic resin (99.6 mg/dL) and silicone soft reliner (126.0 mg/dL) obturator prosthesis (*p* = .043). There was no significant difference in the median of maximum bite force between the acrylic resin (302.0 N) and silicone soft reliner (250.0 N) obturator prosthesis (*p* = .893). Functional limitations domain of the OHIP‐J49 indicated a statistically significant improvement between the acrylic resin and silicone soft reliner obturator prosthesis (*p* = .043).

**Conclusions:**

This study indicated that an obturator relined with soft silicone improved masticatory performance and the OHRQoL post‐maxillectomy.

## 1 INTRODUCTION

Maxillectomy for oral malignant tumors is commonly rehabilitated with acrylic resin obturator prostheses (Keyf, 2001; Rieger, Wolfaardt, Seikaly, & Jha, 2002). However, when the number of remaining teeth is small or the patient is edentulous, or when the cavity is wide, it is often difficult to achieve sufficient functional recovery with obturator prostheses (Gay & King, 1980; Ono, Kohda, Hori, & Nokubi, 2007). Although some clinical researchers reported that silicone soft reliners could be an option for functional recovery after maxillectomy, these have been published as case reports, and do not provide evidence for the efficacy of silicone soft reliner on the obturator prosthesis after maxillectomy (Singh, Kumar, Gupta, & Sikka, 2015; Taira, Yanamoto, Kawasaki, Yamada, & Atsuta, 2007). The silicone soft reliner is a soft denture relining material designed for denture wearers who cannot tolerate conventional hard acrylic denture reline. Although some randomized control trials showed that the application of silicone soft reliner to mandibular complete dentures resulted in significant improvements in the patients' masticatory function compared to conventional hard acrylic denture reline, (Furokawa et al., 2020; Kimoto et al., 2004; Kimoto et al., 2006) few detailed evaluations of the efficacy of silicone soft reliner on maxillary dentures have been performed. Furthermore, in Japan, the use of silicone soft reliner for maxillary dentures is not covered by health insurance; therefore, clinical evaluation has not been performed.

Thus, this single arm prospective interventional study aimed to verify the efficacy of silicone soft reliner on the obturator prosthesis after maxillectomy, by evaluating masticatory performance and quality of life (QoL).

## 2 METHODS

### 2.1 Study design

This was a single arm prospective interventional study verifying the efficacy of silicone soft reliner (GC RELINE II®, GC Co. Ltd, Tokyo, Japan) on the maxillary obturator prosthesis, and was performed in accordance with the 2013 Declaration of Helsinki. The protocol of this study was registered with the Japan Registry of Clinical Trials (jRCT) on September 26, 2019 (jRCTs072190027). Ethical approval was obtained from the Clinical Research Review Board at Nagasaki University (No. CRB19‐011‐1).

### 2.2 Participants and setting

This study included patients wearing the obturator prosthesis following maxillectomy for oral malignant tumors at the Department of Oral and Maxillofacial Surgery, Nagasaki University Hospital between October 2019 and March 2020. The exclusion criteria were as follows: (a) patients with problems in judgement, (b) patients with drug hypersensitivity, who are allergic to silicone material, and (c) patients judged by the investigator to be inappropriate as study subjects. Written informed consent was obtained from each patient.

### 2.3 Intervention

**FIGURE 1:**
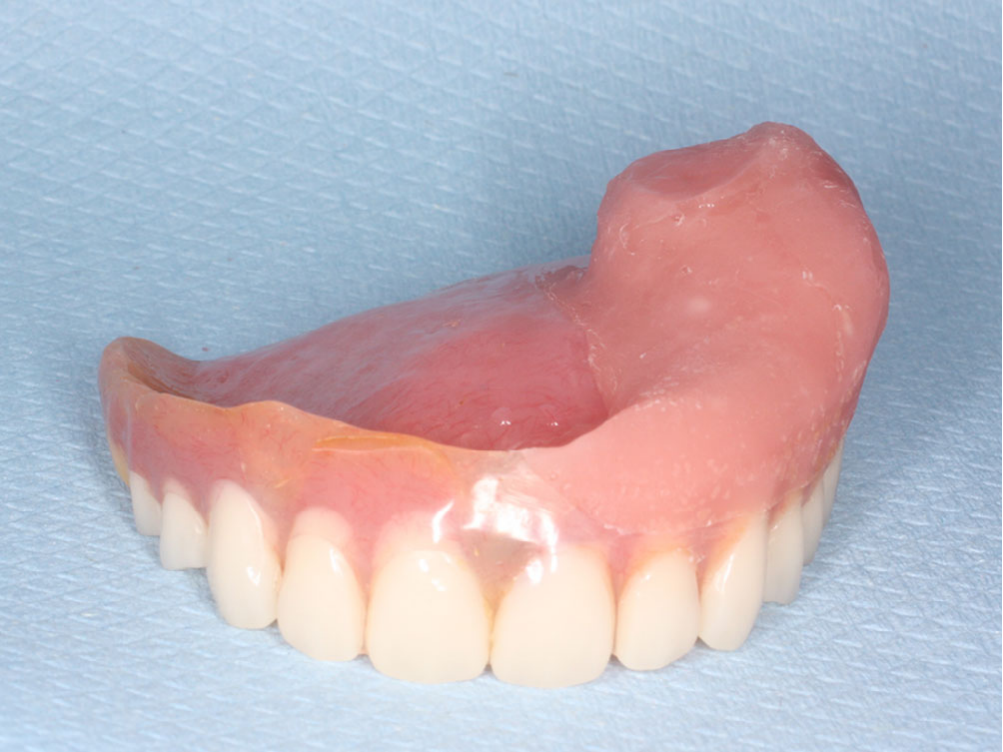
Obturator prosthesis after the application of the silicone soft reliner

The participants wearing newly manufactured acrylic resin obturator prosthesis were evaluated for endpoints after 14 days of continuous prosthesis use. On the same day, a tissue conditioner (Soft‐Liner®, GC Co. Ltd, Tokyo, Japan) was used to make a dynamic impression with the acrylic resin obturator prosthesis. The dynamic impression surface of the acrylic resin obturator prosthesis was poured in type IV dental stone (DF Rock®, San Esu Gypsum Co. Ltd, Hyogo, Japan). After flasking, the impression material was removed from the tissue surface of the obturator prosthesis. The tissue surface was cleaned and painted with adhesive (GC RELINE II® PRIMER, GC Co., Ltd, Tokyo, Japan). After air‐drying, the silicone soft reliner (GC RELINE II®) was applied on the tissue surface of the obturator prosthesis, which was mounted on the gypsum model and pressed in place on an articulator for at least 30 minutes at room temperature. After curing, the excess material was removed, and the edges were smoothened using trimming and finishing wheels (Figure 1). After clinical adjustments, the participants wearing silicone soft relined obturator prosthesis were evaluated for endpoints after 14 days of continuous prosthesis use.

### 2.4 Endpoints and measuring variables

The data was obtained from a comparison of the endpoints of acrylic and silicone soft‐lined prostheses. The primary endpoint was masticatory performance. The secondary endpoints were occlusal performance and oral health‐related QoL (OHRQoL).

Masticatory performance was assessed according to the glucose extraction in the filtrate obtained after chewing gummy jelly (Glucorumm®, GC Co. Ltd, Tokyo, Japan). Participants were asked to chew gummy jelly on their habitual chewing side for 20 s. After chewing, the subjects were asked to hold 10 ml of distilled water in their mouth for a moment, and then spit it into a cup. The cup containing the gummy jelly and the saliva was then filtered, and the filtrate was collected. The glucose concentration in the filtrate (mg/dl) was measured as glucose extraction, using a glucose testing device (Gluco Sensor GS‐II®, GC Co. Ltd, Tokyo, Japan).

Occlusal performance was assessed according to the measurement of maximum bite force (N) using a pressure‐sensitive sheet (Dental Prescale® 50H type R, GC Co. Ltd, Tokyo, Japan). Participants were instructed to bite onto the test sheet as hard as possible for 3 s in the intercuspal position. The sheets were analyzed using analytical Charge‐Coupled Device camera (Occluzer FPD‐707, GC Co. Ltd, Tokyo, Japan).

The questionnaire for analysis of OHRQoL was employed using the Japanese version of the Oral Health Impact Profile (OHIP‐J49) (Yamazaki, Inukai, Baba, & John, 2007). OHIP‐J49 is based on the original 49 items distributed between the following seven domains: functional limitations, pain, psychological discomfort, physical disability, psychological disability, social disability, and handicap. Each item is scored on a Likert‐like scale ranging from zero to four (zero = never, one = hardly ever, two = occasionally, three = fairly often, four = very often) to calculate the total as well as each of the seven domains.

### 2.5 Statistical analysis

Data analyses were performed using the SPSS version 24.0 software (Japan IBM Co., Tokyo, Japan). Descriptive analysis of all variables was expressed as median with interquartile range (IQR). The continuous data between acrylic resin and silicone soft reliner obturator prostheses were assessed by the non‐parametric Wilcoxon signed‐rank test. In all analyses, two‐tailed *p* values <.05 were considered statistically significant.

## 3 RESULTS

### 3.1 Patients characteristics

A total of six patients were enrolled in this prospective interventional study. However, one patient was lost due to difficulty in following directions during examinations. Eventually, this study included five patients, ranging in age from 71 to 88 years, with a median of 74 years. The primary site of all patients was the maxillary gingiva. The demographic data of the two males (40%) and three females (60%) are shown in Table 1. The median (IQR) of the residual teeth was 18.0 (5.0–20.0).

**TABLE 1:** Patients characteristics

Patient number	Gender	Age (years)	Diagnosis	Surgical procedure	Number of residual teeth
1	Male	71	SCC of the maxillary gingiva	Partial maxillectomy	18
2	Male	71	SCC of the maxillary gingiva	Partial maxillectomy	25
3	Female	74	SCC of the maxillary gingiva	Partial maxillectomy	5
4	Female	83	SCC of the maxillary gingiva	Subtotal maxillectomy	20
5	Female	88	SCC of the maxillary gingiva	Partial maxillectomy	4

Abbreviation: SCC, squamous cell carcinoma.

### 3.2 Masticatory performance

**FIGURE 2:**
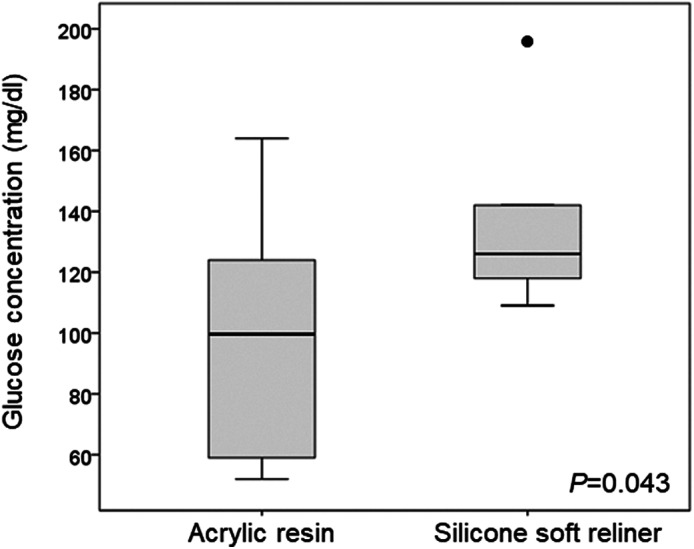
Change of masticatory performance after the application of the silicone soft reliner. Box plots are displayed for glucose concentration (mg/dL). The 25th and 75th percentiles are represented by the upper and lower margins, and median values by horizontal black lines. Whiskers represent the maximum value (top) and the minimum value (bottom) of the dataset. Outliers are represented by a dot

The median (IQR) of glucose concentration indicated a statistically significant improvement between the acrylic resin [99.6 mg/dl (59.0–124.0)] and silicone soft reliners [126.0 mg/dl (118.0–142.0)] obturator prosthesis (*p* = .043) (Figure 2).

### 3.3 Occlusal performance

**FIGURE 3:**
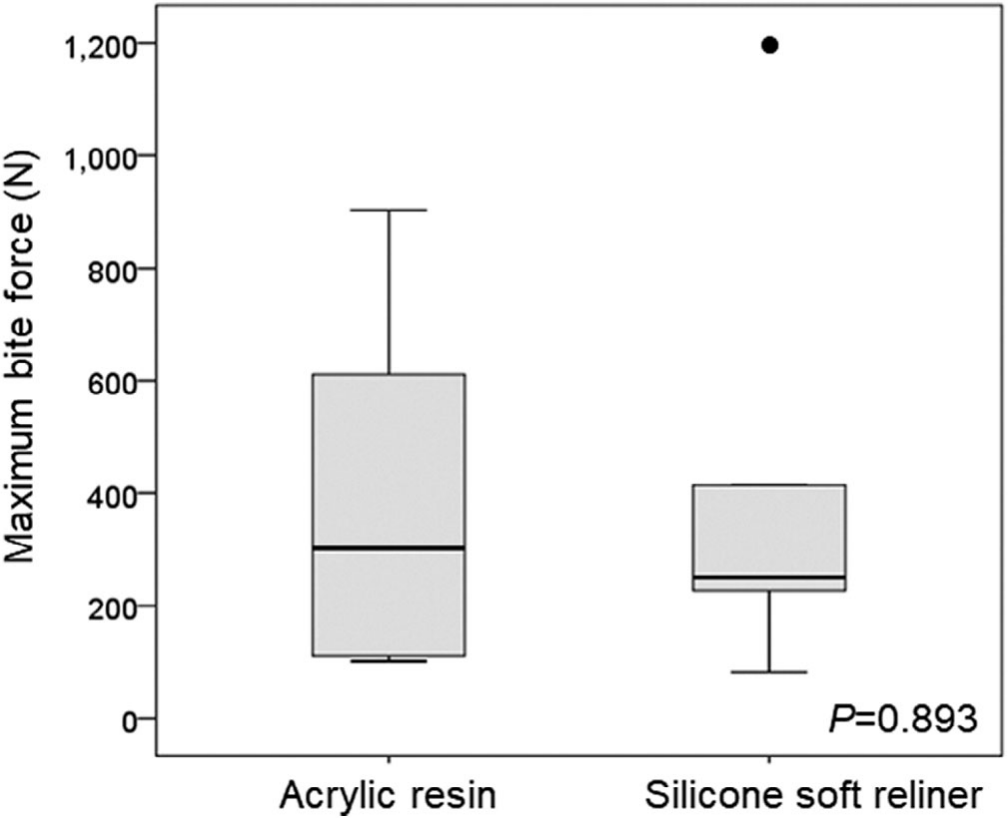
Change of occlusal performance after the application of the silicone soft reliner. Box plots are displayed for maximum bite force (N). The 25th and 75th percentiles are represented by the upper and lower margins, and median values by horizontal black lines. Whiskers represent the maximum value (top) and the minimum value (bottom) of the dataset. Outliers are represented by a dot

There was no statistical difference in the median (IQR) of maximum bite force between the acrylic resin [302.0 N (111.0–611.0)] and the silicone soft reliner [250.0 N (226.9–414.3)] obturator prosthesis (*p* = .893) (Figure 3).

### 3.4 OHRQoL

Functional limitations domain of the OHIP‐J49 indicated a statistically significant improvement between the acrylic resin and silicone soft reliner obturator prosthesis (*p* = .043) (Table 2). The pain domain was not statistically significant, but tended to improve after the application of the silicone soft reliner.

**TABLE 2:** Median of OHIP‐J49 domain sum scores for acrylic resin and silicone soft reliner obturator prostheses

Variables	Acrylic resin median (IQR)	Silicone soft reliner median (IQR)	*p* value
Total (49 items)	47.0 (6.0–60.0)	13.0 (4.0–26.0)	.225
Domains			
Functional limitations (9 items)	14.0 (5.0–15.0)	4.0 (1.0–5.0)	.043
Pain (9 items)	11.0 (1.0–14.0)	0 (0–3.0)	.066
Psychological discomfort (5 items)	4.0 (0–10.0)	2.0 (1.0–2.0)	.461
Physical disability (9 items)	13.0 (0–16.0)	3.0 (1.0–7.0)	.273
Psychological disability (6 items)	2.0 (0–2.0)	0 (0–7.0)	1.000
Social disability (5 items)	0 (0–1.0)	0 (0–1.0)	1.000
Handicap (6 items)	1.0 (0–6.0)	0 (0–6.0)	.581

Abbreviations: IQR, interquartile range; OHIP‐J49, Japanese version of Oral Health Impact Profile.

## 4 DISCUSSION

In this prospective interventional study, the authors aimed to verify the efficacy of silicone soft reliner on the obturator prosthesis after maxillectomy. Among the various factors that affect masticatory function in the maxillary obturator prostheses, surgical maxillectomy for malignant tumors has been reported as one of the most important factors (Ono et al., 2007; Taira et al., 2007). The treatment after maxillectomy for malignant tumors includes rehabilitation with obturator prosthesis, or reconstructive surgery with autogenous tissue grafts. The choice of treatment after maxillectomy depends on each case, and the location and extent of the defect does not always correlate with the method of rehabilitation (Dos Santos et al., 2018). The advantages of obturator prosthesis after maxillectomy over autogenous tissue reconstruction are that the surgical site recurrence may be easily detected, the surgical invasion may be minimal, and that functional recovery can be obtained by acquiring occlusion early after surgery (Ali, Khalifa, & Alhajj, 2018; Dos Santos et al., 2018). On the other hand, it has a problem of nasal leakage due to incomplete separation of the maxillary sinus from the oral cavity. Moreover, some authors report that physical injury and pain caused by the direct contact of acrylic resin to the mucosal surface reduce the QoL (Ali et al., 2018; Ono et al., 2007; Singh et al., 2015). Therefore, further modification of the maxillary obturator prosthesis may contribute to functional recovery and QoL improvement in patients who have undergone maxillectomy for malignant tumor.

A recent systematic review showed that soft silicone reliner provided denture wearers with increased masticatory function compared to conventional acrylic resin materials (Palla, Karaoglani, Naka, & Anastassiadou, 2015). Some authors reported that the application of silicone soft reliner to mandibular complete dentures resulted in significant improvements in the patients' masticatory performance and OHRQoL compared to conventional hard acrylic denture reline (Furokawa et al., 2020; Hayakawa, Hirano, Takahashi, & Keh, 2000; Kar, Tripathi, & Fatima, 2019; Kimoto et al., 2004; Kimoto et al., 2006; Pisani et al., 2012). However, there are few reports regarding the application of a silicone soft reliner to maxillary obturator prosthesis, (Singh et al., 2015; Taira et al., 2007) and its verification has not been done at all.

In this study, the masticatory performance evaluated by glucose concentration significantly improved after the application of the silicone soft reliner compared with acrylic resin. (Hayakawa et al., 2000) and (Kar et al., 2019) reported that applying silicone soft reliner to the mandibular complete denture increased masticatory performance by approximately 30%. Although the method of assessing masticatory performance was different, the results of this study were similar to those of the maxillary obturator prostheses but not to those of mandibular complete dentures. However, occlusal performance assessed by maximum bite force using the Dental Prescale® system was not significantly different between acrylic resin and the silicone soft reliner. Previous reports regarding mandibular complete dentures have not found the application of silicone soft reliner affecting maximum occlusal force (Kimoto et al., 2006; Murata, Taguchi, Hamada, Kawamura, & McCabe, 2002). The results of the present study showed that there was no significant difference, but the maximum occlusal force tended to decrease by the application of silicone soft reliner to maxillary obturator prostheses. These results suggest that applying silicone soft reliner to the supporting tissue defect caused by maxillectomy does not increase the occlusal performance, but improves masticatory performance.

The present study investigated OHRQoL after applying silicone soft reliner to the maxillary obturator prosthesis in patients with maxillectomy for malignant tumor. The OHIP‐J49 was adopted as an evaluation method for OHRQoL in this study. The OHIP was developed in Australia, but is gaining popularity in other countries, and has been translated into Japanese to prove its good reliability and validity (Yamazaki et al., 2007). In this study, the silicone soft reliner used for relining maxillary obturator prosthesis decreased the total scores of functional limitations domain more than acrylic resin. It was speculated that the domain of functional limitations was significantly improved after applying the silicone soft reliner due to the improvement in masticatory performance. Additionally, although data is not shown in the results, the presence of nasal leakage was assessed by whether or not water leaked into the nasal cavity during swallowing. Nasal leakage was found in three of five participants with acrylic resin obturator prosthesis, while there was no presence of nasal leakage with the silicone soft reliner obturator prosthesis. This result may also contribute to the improvement in the domain of functional limitations. Moreover, the OHIP‐J49 results showed that the other domains also tended to improve after applying the silicone soft reliner. The reason for such unsatisfactory results may be the small number of participants.

This study had several limitations. The study design was single armed, the sample size was small, and the observation interval was short (14 days). It is possible that appropriate statistical analysis could not be performed due to the small number of participants. However, to the best of our knowledge, this is the first study to confirm the efficacy of silicone soft reliner on the obturator prosthesis after maxillectomy, by evaluating masticatory function and OHRQoL. In the future, longitudinal prospective studies with a large number of participants are required.

## 5 CONCLUSION

This study showed that applying silicone soft reliner to the maxillary obturator prosthesis in patients with maxillectomy for malignant tumor improved masticatory performance and the OHRQoL. Therefore, its application to the maxillary obturator prosthesis is clinically beneficial.

## CONFLICTS OF INTEREST

The authors have conflicts of interest to disclose concerning the study. This study was supported by the research materials from GC Co. Ltd, Tokyo, Japan.
